# Supplementary figures and images for: Ecological ubiquity and phylogeny drive nestedness in phages–bacteria networks and shape the bacterial defensome
Source: PLoS Pathog. 2025 Dec 30;21(12):e1013428. doi: 10.1371/journal.ppat.1013428 (PMC12782414; doi:10.1371/journal.ppat.1013428)

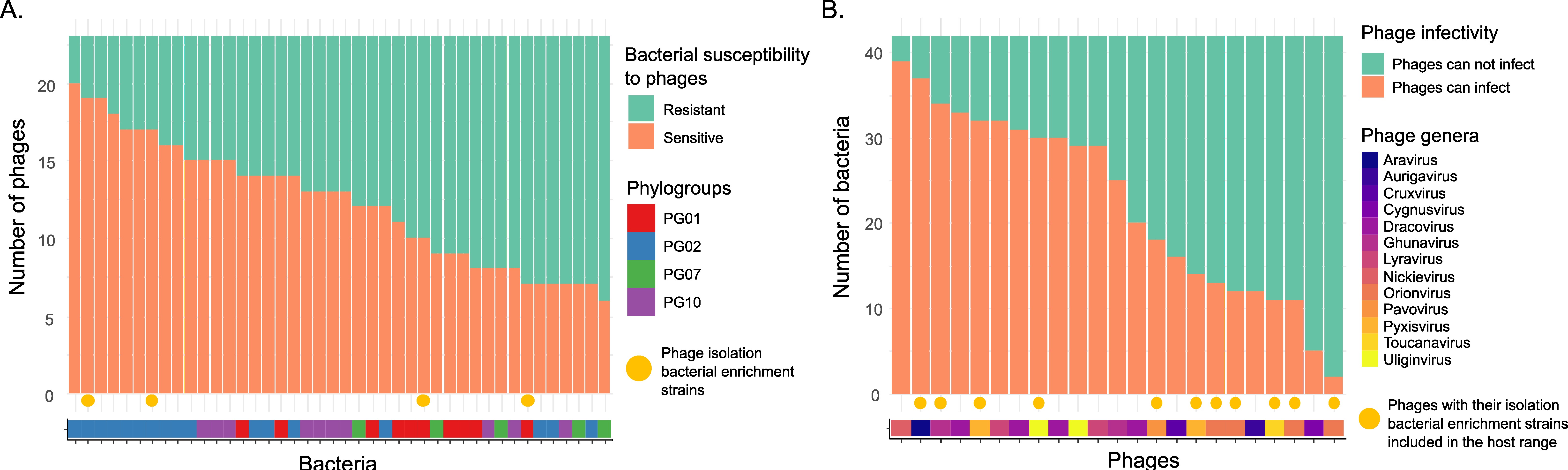

Supplement: S1 Fig — (A) Number of phages to which bacteria are sensitive or resistant, with the phylogeny of the strains indicated at the bottom of the histogram. (B) Number of bacteria infected or not by the phages, with the taxonomy of the phages listed at the bottom of the histogram. Values are derived from the binary inhibition matrix. (JPG) [file ppat.1013428.s001.jpg]

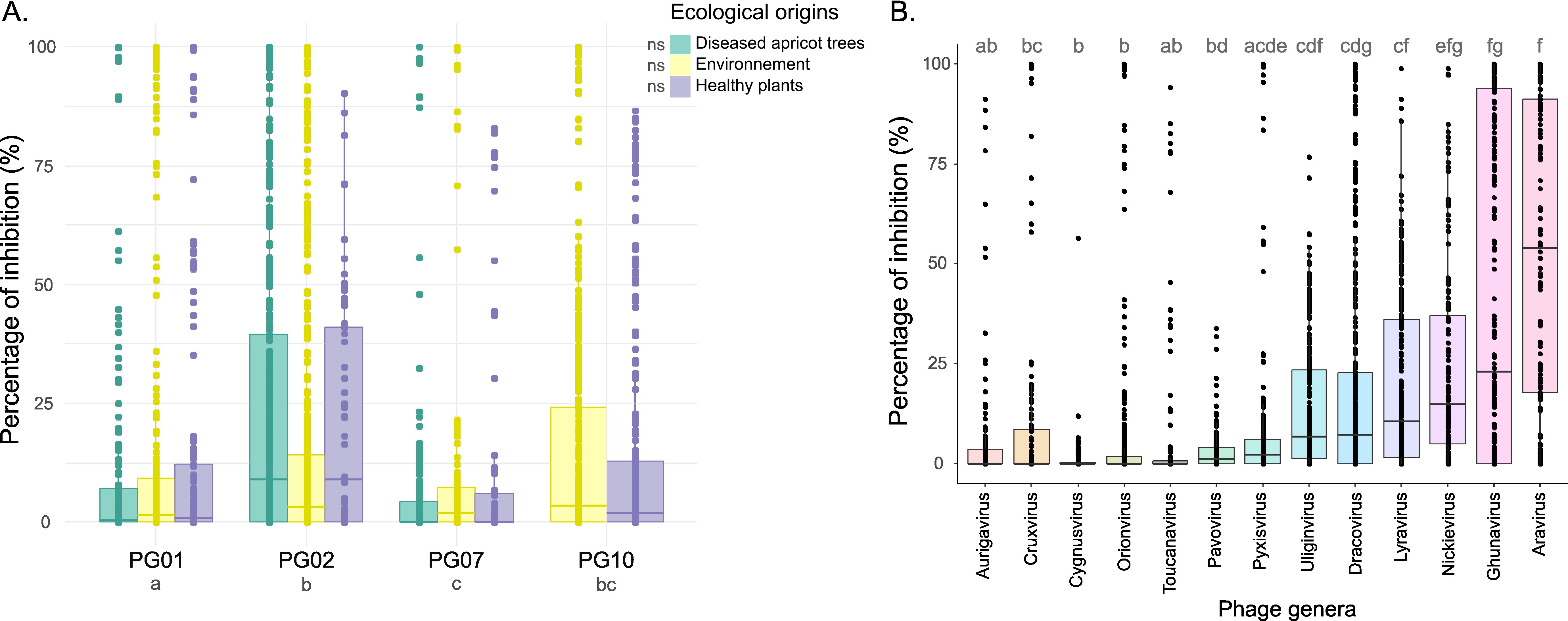

Supplement: S2 Fig — (A) Bacterial percentage of inhibition by phages across bacterial phylogroups and ecological origins. Significance letters indicate results from the PERMANOVA test and associated pairwise comparisons. (B) Bacterial inhibition percentage of different phage genera. Significance letters represent results from a GLMM with a Gamma regression distribution and random effect (phage, bacteria, and the replicate imbricated in each of the phage-bacteria interactions), and post hoc pairwise comparisons performed using Tukey’s test. (JPG) [file ppat.1013428.s002.jpg]

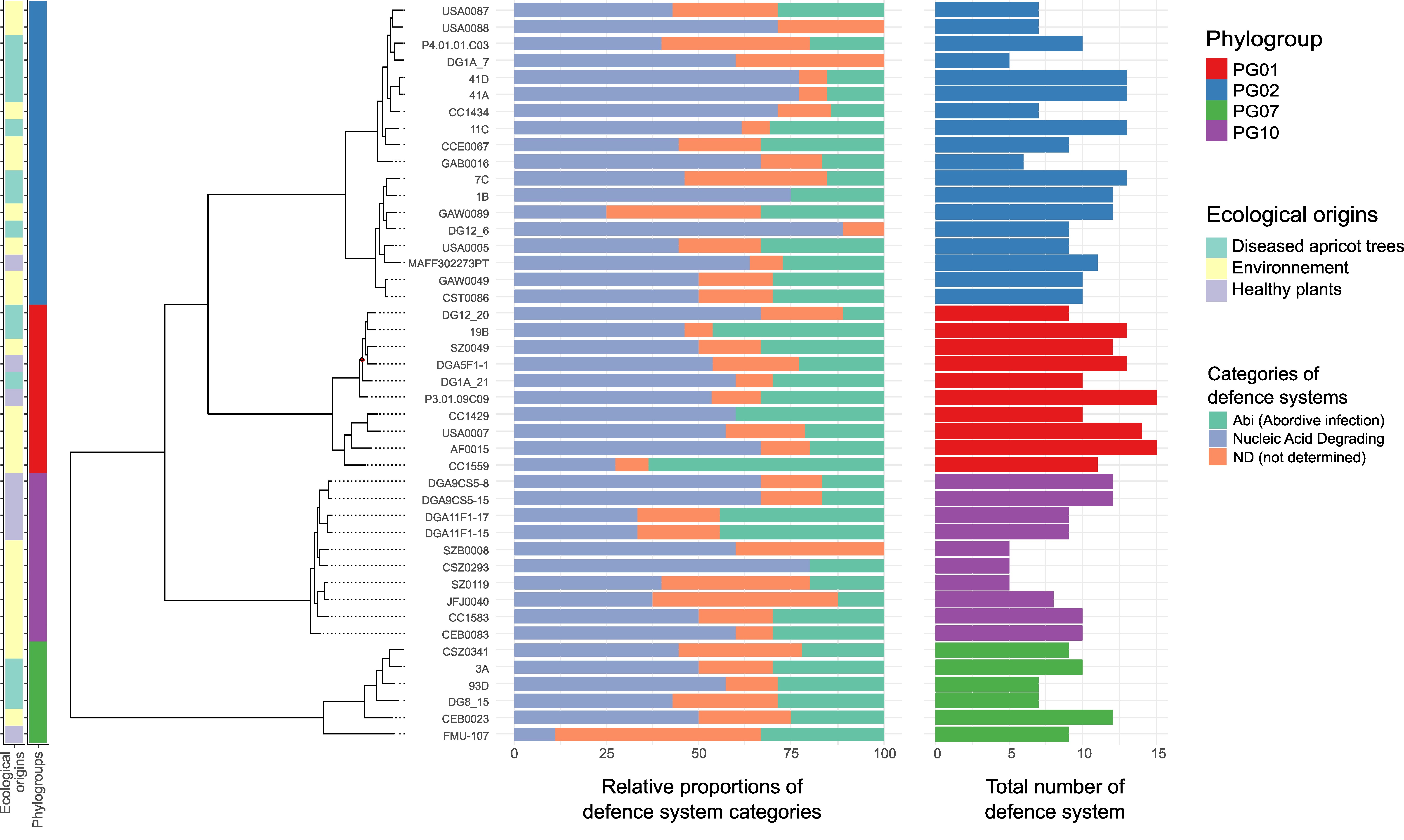

Supplement: S3 Fig — On the left, a phylogenetic tree based on the core genomes of the bacterial strains is shown, with corresponding phylogroups indicated in the adjacent column. (JPG) [file ppat.1013428.s003.jpg]

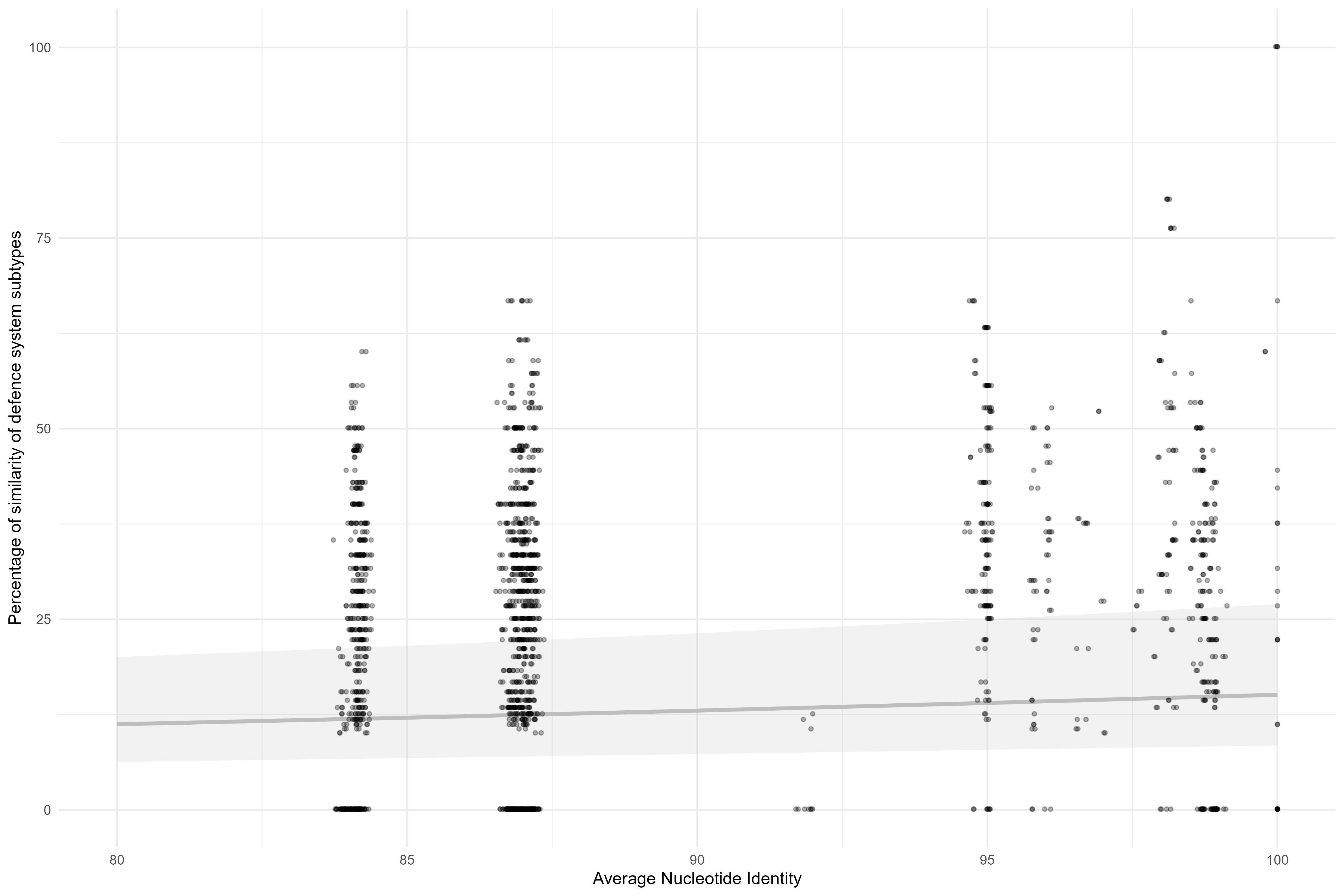

Supplement: S4 Fig — Transparent grey dots represent individual observations (darker areas indicate overlap). The overall trend is shown by the grey line, which represents the mean prediction from the GLMM accounting for random effects associated with bacterial strains. The shaded ribbon indicates the 95% confidence interval of the model’s marginal predictions. (JPG) [file ppat.1013428.s004.jpg]

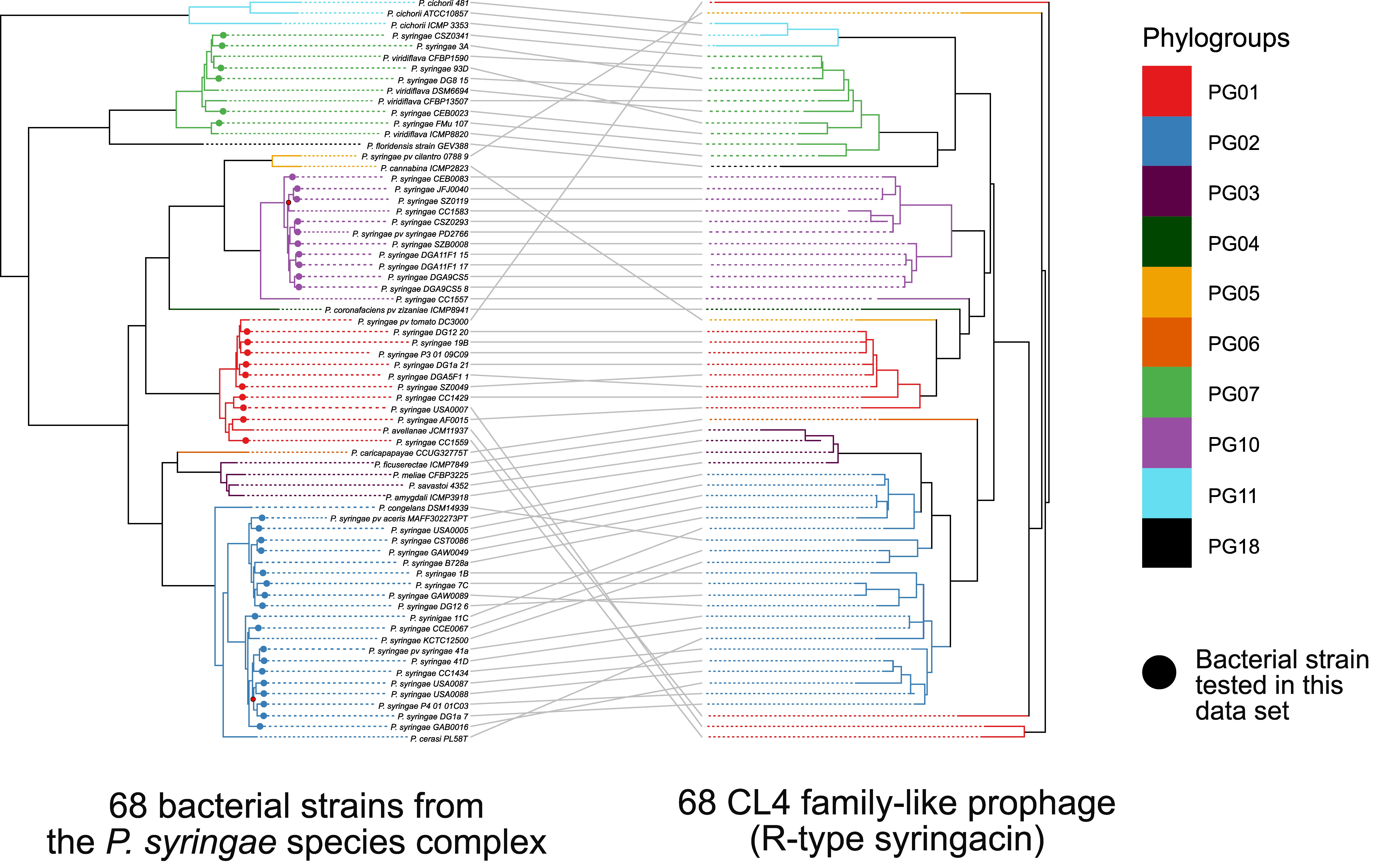

Supplement: S5 Fig — Co-phylogenetic analysis between 68 strains of the P. syringae species complex (left), represented by a maximum likelihood phylogenetic tree based on their core-genomes, and their associated 68 CL4 family-like prophages (right), represented by a proteomic tree. The 44 bacterial strains included in this study are marked with dots at the tips of the bacterial tree. Phylogroups and prophage-carrying strains are colour-coded accordingly. Two nodes with a maximum likelihood support value below 0.90 are marked with red dots. (JPG) [file ppat.1013428.s005.jpg]

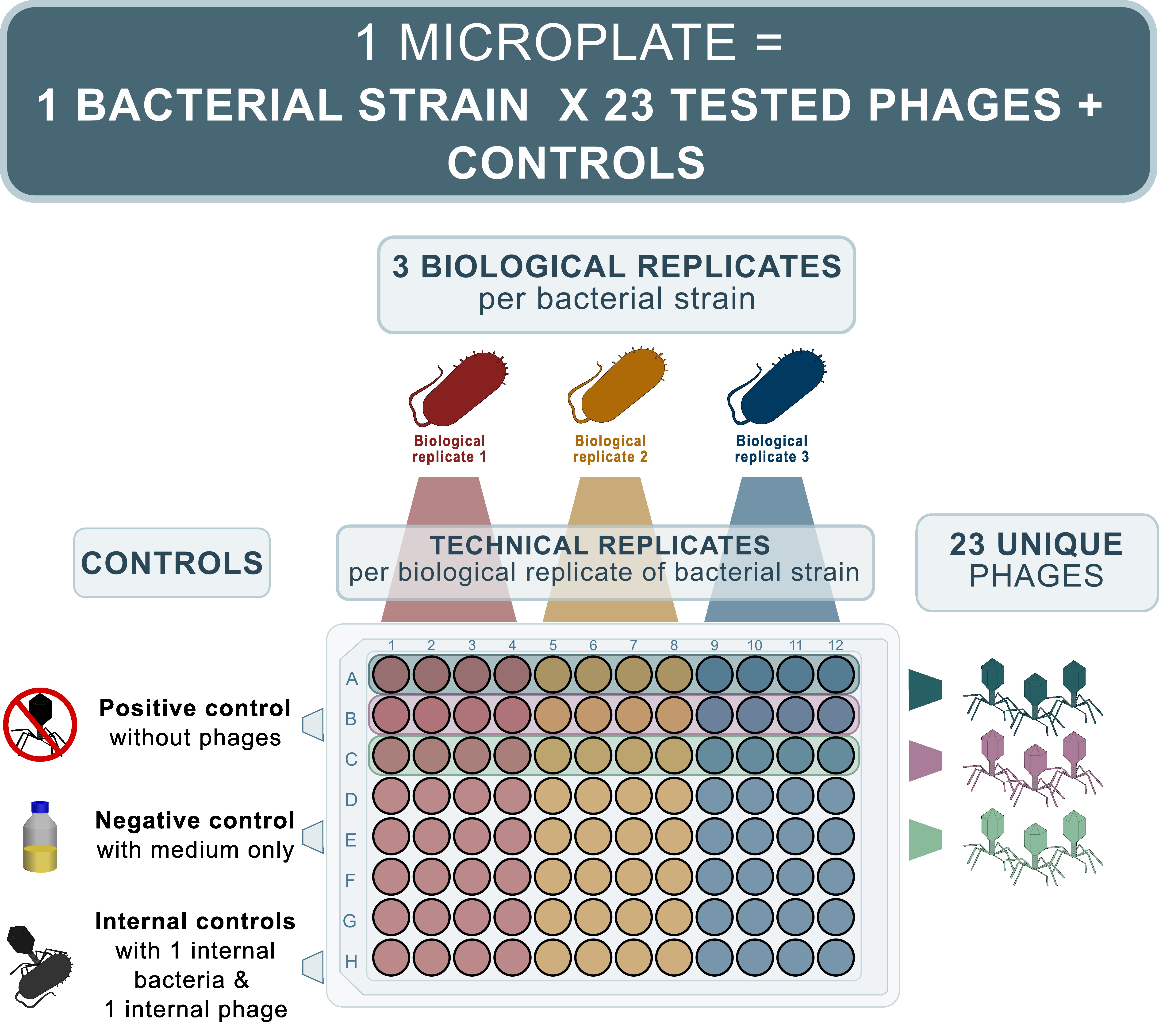

Supplement: S6 Fig — Each biological replicate per bacterial strain is represented by a different colour, and each row corresponds to the technical replicates within that biological replicate. For clarity, only 3 of the 23 tested phages are shown in this schematic, which does not depict the randomisation of phages across plates. Three types of internal controls are included: positive controls with bacteria only, negative controls with medium only, and internal reference controls using the bacterial strain P1.01.01B03 (growth shown in S7B Fig) and the internal phage Ghuch01, which consistently inhibits this strain (see S7C Fig). Icons were obtained from open-source resources (microplate: https://commons.wikimedia.org/wiki/File:96-Well_plate.svg; phage: https://openclipart.org/detail/62785/virus; bacteria: https://openclipart.org/detail/221334/lamarque-disease; bottle of media: https://openclipart.org/detail/128449/reagent-bottle). (JPG) [file ppat.1013428.s006.jpg]

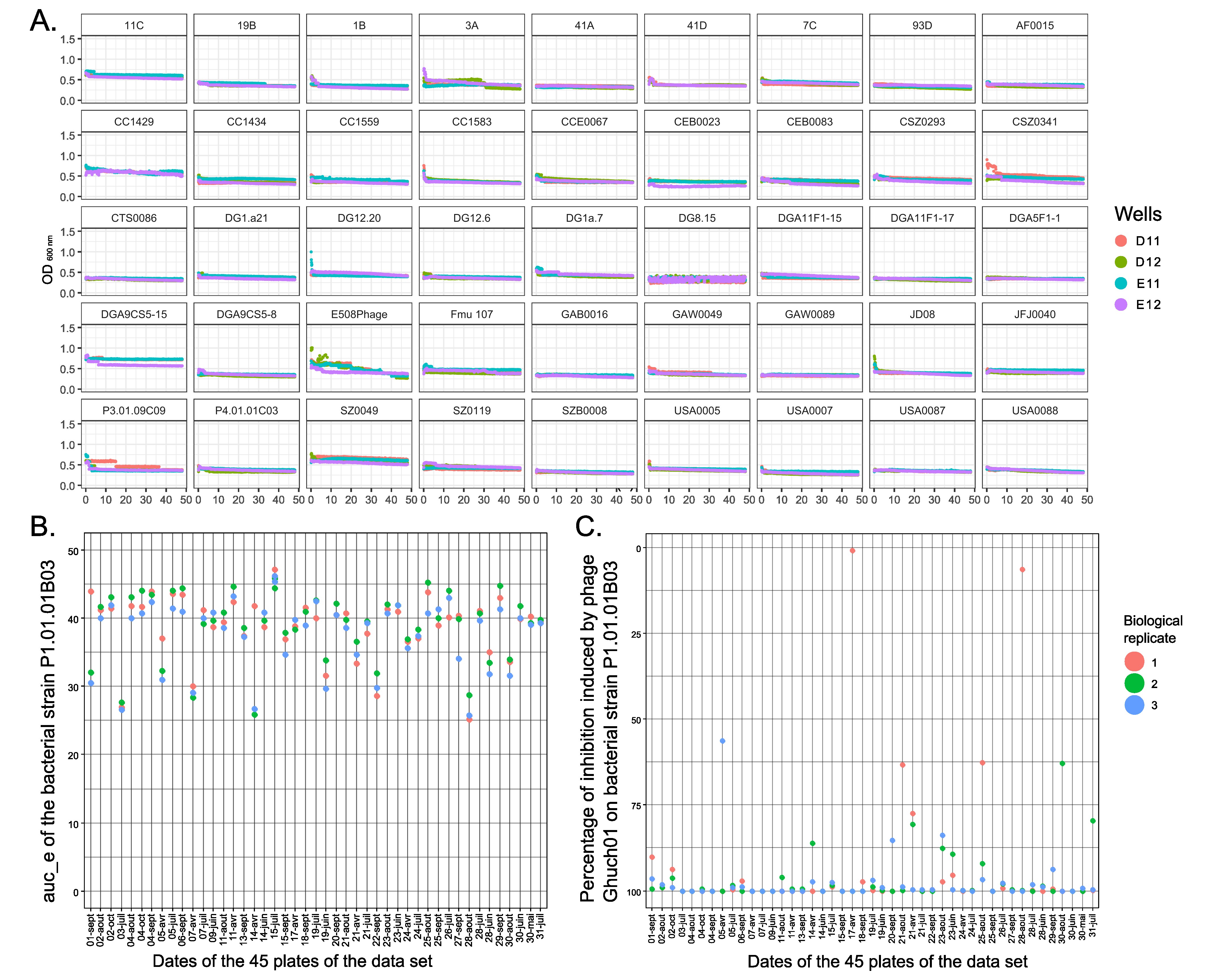

Supplement: S7 Fig — (A) Analysis of culture medium controls by plate (labelled by bacteria). (B) Area under the empirical curve (auce) of the internal bacterial control, P. syringae strain P1.01.01B03. (C) Inhibition of the internal phage control Ghuch01 on the internal bacterial control (%). (JPG) [file ppat.1013428.s007.jpg]

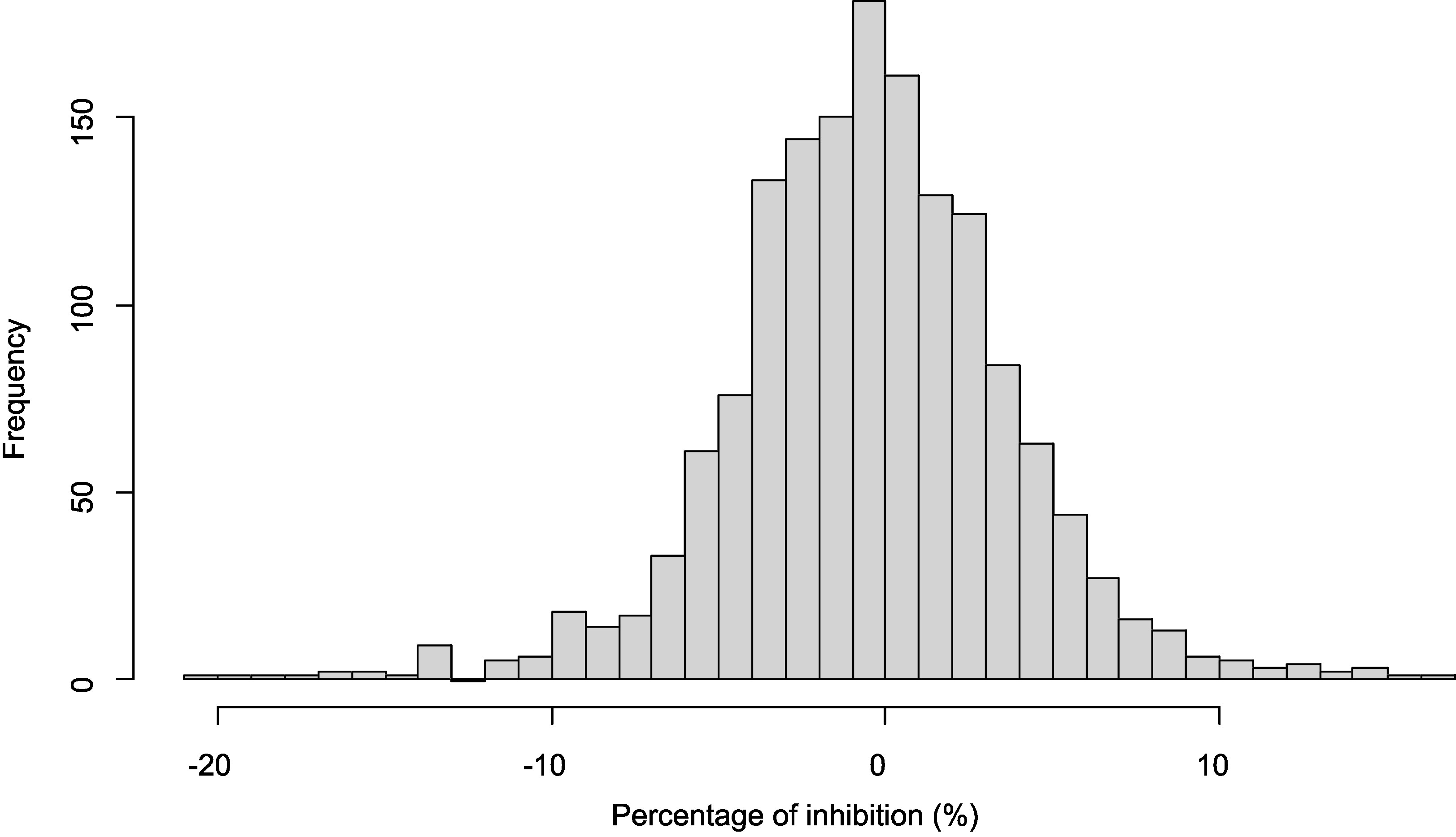

Supplement: S8 Fig — (JPG) [file ppat.1013428.s008.jpg]

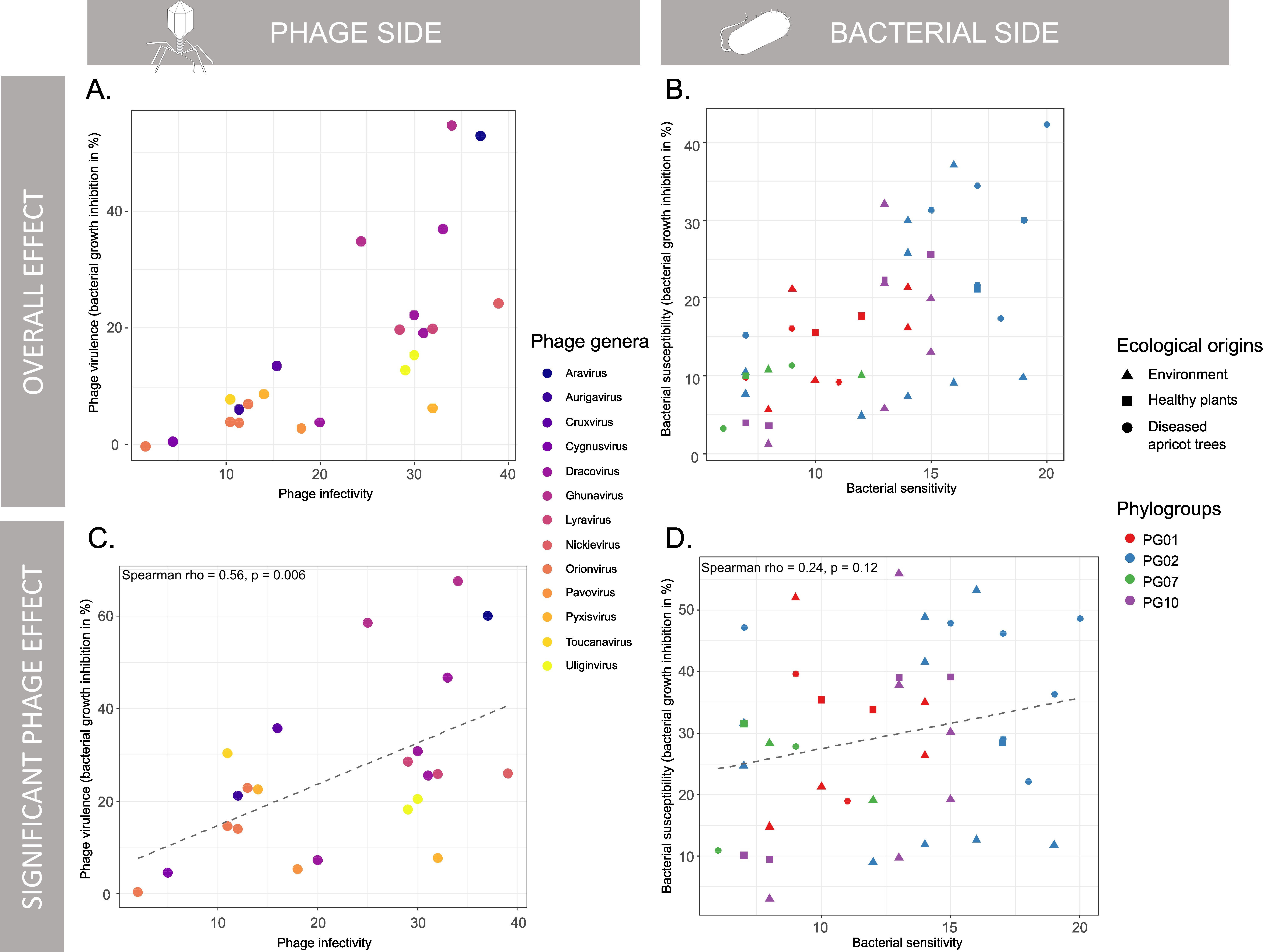

Supplement: S9 Fig — (A, C) Phage perspective: mean percentage of inhibition per phage (y-axis) is plotted against the number of bacterial strains each phage significantly infected (x-axis). Colours indicate phage genera. (B, D) Bacterial perspective: mean percentage of inhibition per bacterial strain (y-axis) is plotted against the number of phages significantly infecting each strain (x-axis). Colours represent bacterial phylogroups, and shapes indicate ecological niches. (A, B) Analyses based on overall inhibition values. (C, D) Analyses restricted to significant phage–bacterium interactions. Spearman’s rho and associated p-values are reported for panels (C) and (D). Icons were obtained from open-source resources (phage: https://openclipart.org/detail/62785/virus; bacteria: https://openclipart.org/detail/221334/lamarque-disease). (JPG) [file ppat.1013428.s009.jpg]

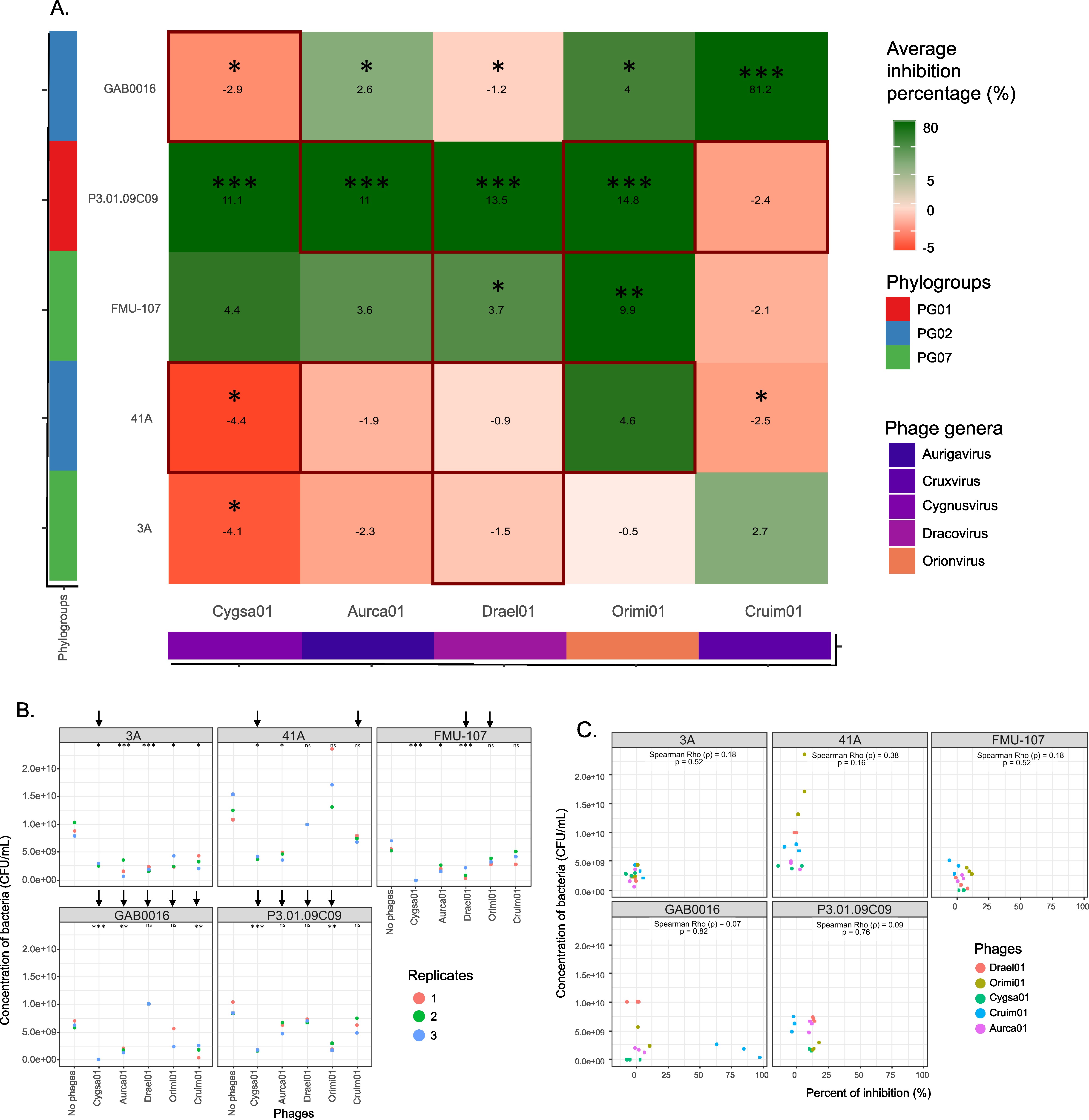

Supplement: S10 Fig — Generally, bacterial populations decreased in the presence of phages. In 17% of observed phage-bacteria combinations, a “stimulatory” effect was identified, with inhibition values reaching up to -40%. Five phages showing strong stimulatory effect and five bacteria showing significant stimulation were chosen to test the reproducibility of the stimulation effect in subsequent experiments. (A) Matrix of mean inhibition percentages for the five P. syringae strains (rows) and five phages (columns), with their phylogenetic and taxonomic affiliations indicated by colour. The number of stars indicates the number of replicates showing a significantly inhibiting or stimulating percentage. Of ten initially identified significant stimulating interactions (delimited by red lines), only three were confirmed in subsequent experiments. (B) Size of the five bacterial populations in response to the phages. Stars correspond to significant differences from the control (no phage) based on the results of the GLM model, family = Gamma. Arrows indicate interactions with at least one of the replicates showing a significant inhibition or stimulation percentage. Out of the three significant interactions of stimulatory phenotype, only 2 phage-bacteria interactions resulted in slightly higher bacterial densities in the presence of phages compared to phage-free conditions, though this difference was not statistically significant. (C) Spearman correlation between bacterial concentration and inhibition percentage, by bacterium and phage. No correlation was significant and bacterial population size only poorly explains the inhibition percentages. (JPG) [file ppat.1013428.s010.jpg]

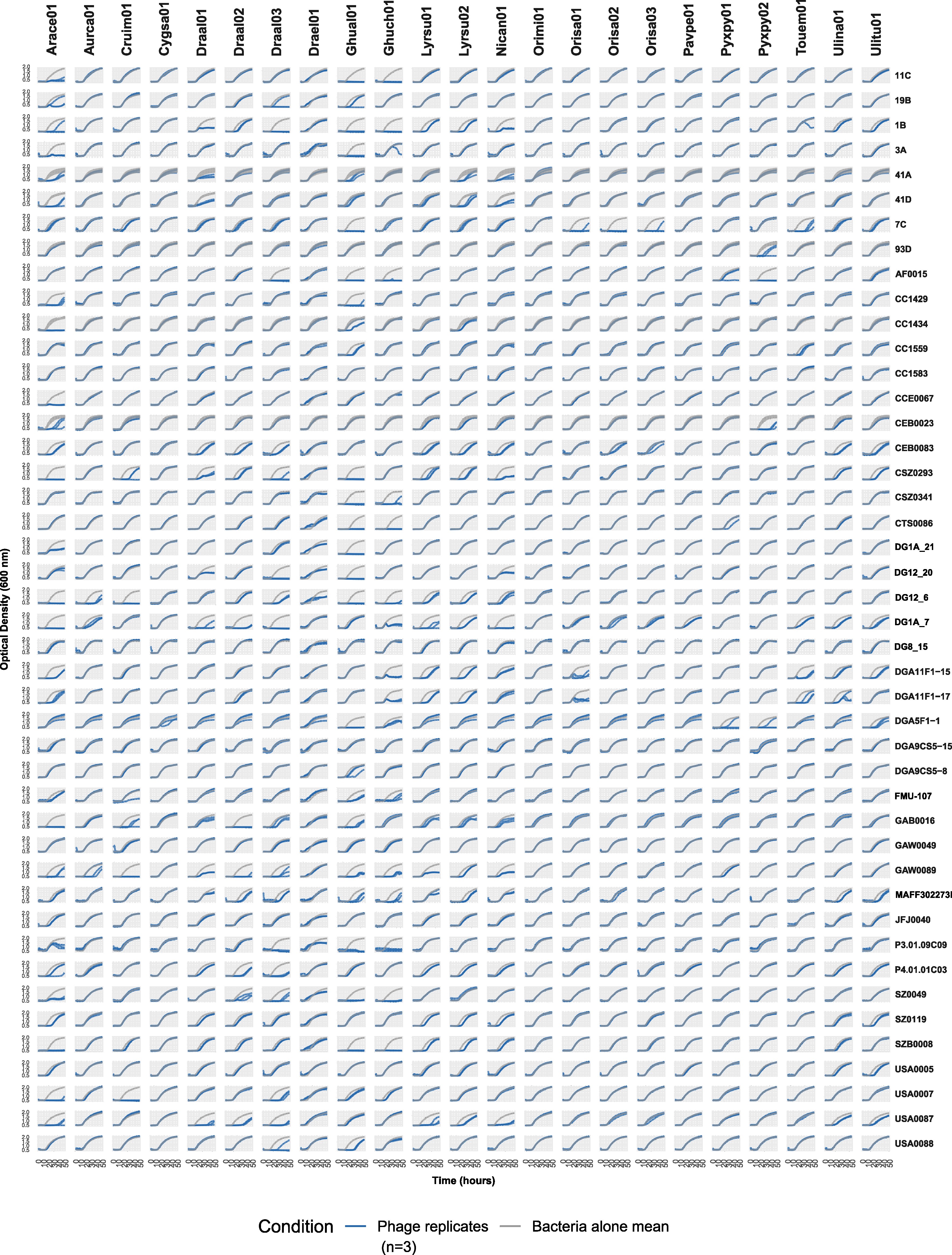

Supplement: S11 Fig — Optical density (OD) is plotted over time (hours) for each phage–bacteria interaction, with phages (n = 23) in columns and bacterial strains (n = 44) in rows. Grey curves represent the average growth of bacteria alone for each phage–bacteria pair. Blue curves show individual replicates for each interaction (n = 3), illustrating the variability in bacterial response to phage infection. (JPG) [file ppat.1013428.s011.jpg]
